# Prevalence, Predictors, and Same Day Treatment of Positive VIA Enhanced by Digital Cervicography and Histopathology Results in a Cervical Cancer Prevention Program in Cameroon

**DOI:** 10.1371/journal.pone.0157319

**Published:** 2016-06-09

**Authors:** Geneva A. DeGregorio, Leslie S. Bradford, Simon Manga, Pius M. Tih, Richard Wamai, Rebecca Ogembo, Zacharie Sando, Yuxin Liu, Constance Schwaiger, Sowmya R. Rao, Karen Kalmakis, Lisa Kennedy Sheldon, Kathleen Nulah, Edith Welty, Thomas Welty, Javier Gordon Ogembo

**Affiliations:** 1 Department of Obstetrics and Gynecology, University of Massachusetts Medical School, Worcester, MA, United States of America; 2 Cameroon Baptist Convention Health Services, Bamenda, North West Region, Cameroon; 3 Department of African American Studies, Northeastern University, Boston, MA, United States of America; 4 Graduate School of Nursing, University of Massachusetts Medical School, Worcester, MA, United States of America; 5 Yaoundé Gyneco-Obstetrics and Pediatric Hospital, Yaoundé, Centre Region, Cameroon; 6 Department of Pathology, University of Massachusetts Medical School, Worcester, MA, United States of America; 7 School of Nursing, University of Massachusetts Amherst, Amherst, MA, United States of America; 8 Department of Surgery, Boston University School of Medicine, Boston, MA, United States of America; 9 Department of Nursing, University of Massachusetts Boston, Boston, MA, United States of America; 10 Department of Medicine, University of Massachusetts Medical School, Worcester, MA, United States of America; State University of Maringá/Universidade Estadual de Maringá, BRAZIL

## Abstract

**Background:**

In 2007, the Cameroon Baptist Convention Health Services (CBCHS) implemented a screen-and-treat cervical cancer prevention program using visual inspection with acetic acid enhanced by digital cervicography (VIA-DC).

**Methods:**

We retrospectively analyzed 46,048 medical records of women who received care through the CBCHS Women’s Health Program from 2007 through 2014 to determine the prevalence and predictors of positive VIA-DC, rates of same day treatment, and cohort prevalence of invasive cervical cancer (ICC).

**Results:**

Of the 44,979 women who were screened for cervical cancer, 9.0% were VIA-DC-positive, 66.8% were VIA-DC-negative, 22.0% were VIA-DC-inadequate (normal ectocervix, but portions of the transformation zone were obscured), and 2.2% were VIA-DC-uncertain (cervical abnormalities confounding VIA-DC interpretation). Risk factors significantly associated with VIA-DC-positive screen were HIV-positivity, young age at sexual debut, higher lifetime number of sexual partners, low education status and higher gravidity. In 2014, 31.1% of women eligible for cryotherapy underwent same day treatment. Among the 32,788 women screened from 2007 through 2013, 201 cases of ICC were identified corresponding to a cohort prevalence of 613 per 100,000.

**Conclusions:**

High rate of VIA-DC-positive screens suggests a significant burden of potential cervical cancer cases and highlights the need for expansion of cervical cancer screening and prevention throughout the 10 regions of Cameroon. VIA-DC-inadequate rates were also high, especially in older women, and additional screening methods are needed to confirm whether these results are truly negative. In comparison to similar screening programs in sub-Saharan Africa there was low utilization of same day cryotherapy treatment. Further studies are required to characterize possible program specific barriers to treatment, for example cultural demands, health system challenges and cost of procedure. The prevalence of ICC among women who presented for screening was high and requires further investigation.

## Introduction

Invasive cervical cancer (ICC) is the leading cause of cancer mortality among women in sub-Saharan Africa (SSA) with approximately 99,000 new cases and 57,400 deaths in 2012 [1, 2]. The high burden of ICC in SSA is due to low awareness about screening, limited healthcare infrastructure and a paucity of cost-effective screening programs [3]. In addition, the expanding number of women living with human immunodeficiency virus (HIV) and human papillomavirus (HPV) co-infection is thought to have had a significant impact on the increasing number of ICC cases [4]. Approximately 70% of the 36.9 million people living with HIV globally are in SSA [5], and Cameroon has the highest HIV prevalence in West and Central Africa [6]. Furthermore, owing to certain increased susceptibilities, women make up the majority of the HIV seropositive population in Africa [7], and nearly 40% of HIV seropositive women without visible cervical abnormalities test positive for HPV infection, thus increasing their risk for developing cervical intraepithelial neoplasia (CIN) or ICC [8].

Despite their relative increased risk, less than 19% of women in developing countries have been screened for CIN even once in their lifetime [9]. This is a missed opportunity as screening a woman with visual inspection with acetic acid (VIA) just once between the ages of 30 and 49 has been shown to decrease her lifetime risk of developing ICC by 26% [10]. Many developing countries report rates of VIA-positivity in order to reflect burden of pre-cancerous lesions [11–26]; however, the data regarding VIA-positivity in Cameroon is limited. Only two small studies have addressed this issue, and report VIA-positive rates of 12.9% and 21.7% [27, 28]. Additionally, ICC is reported as the second leading cause of cancer mortality and incidence among women in Cameroon by GLOBOCAN 2012 from the International Agency for Research on Cancer (IARC) (17.5 deaths per 100,000 per year and 30.0 cases per 100,000 per year) [2]. However, this may be an underestimation due to the lack of specific country-level data on cervical cancer screening and the absence of a national cancer registry [1, 9, 29]. The quality of data included in these worldwide analyses varies by country and in an evaluation of country-specific GLOBOCAN 2012 data, Cameroon received a score of E6 on a scale of excellent (A1) to poor (G6) [30]. As such there is a need for more data describing rates of VIA-positivity and ICC in Cameroon.

Cervical cancer screening is not provided routinely in the public health system utilized by the majority of the Cameroonian population [31]. Therefore there is an urgent need for large scale, sustainable cervical cancer screening programs throughout the country. In 2007, the Cameroon Baptist Convention Health Services (CBCHS), a faith-based healthcare organization, founded the Women’s Health Program (WHP) and began to provide World Health Organization (WHO)-endorsed screen-and-treat cervical cancer screening using a fee-for-service model [32]. Utilizing a clinical database of 44,979 women screened for cervical cancer by the CBCHS WHP from 2007 through 2014, we (i) describe the prevalence and predictors of positive visual inspection with acetic acid enhanced by digital cervicography (VIA-DC) screen, (ii) document rates of same day treatment in women with pre-cancerous lesions and (iii) describe cohort prevalence of ICC in a large scale screening program in Cameroon.

## Materials and Methods

### Procedure

The Institutional Review Boards at University of Massachusetts Medical School (UMMS) and Cameroon Baptist Convention Health Services approved this project. The informed consent requirement was waived for this retrospective chart review. The patient records were de-identified and analyzed anonymously.

The WHP procedure for cervical cancer screening was modeled after the Cervical Cancer Prevention Program in Zambia (CCPPZ), a regional leader in implementing screening programs based on VIA-DC [14]. This technique employs a commercial brand digital camera projecting magnified, real-time images of the cervix onto a television monitor visible to both patient and provider. Permanent photographs of the cervix (cervicographs) are then stored to assist with quality control and patient/provider education [33]. All screening done by WHP was performed by nurse clinicians according to standard protocol as outlined by the IARC [34]. The procedure began with examination of the external genitalia, followed by a sterile speculum exam of the cervix and upper vagina. Diluted 3–5% acetic acid was then applied to the cervix for 2 minutes, after which visual inspection was performed to assess for acetowhite changes. Cervicographs were taken both before and after application of acetic acid. Following VIA-DC, visual inspection with Lugol’s iodine (VILI-DC) was also performed and another cervicograph was obtained. Results of VIA-DC and VILI-DC were classified as negative, positive, uncertain or inadequate. Detection of a distinct opaque acetowhite lesion on the cervix was considered VIA-DC-positive. Results classified as VIA-DC-uncertain included those with non-neoplastic cervical abnormalities confounding VIA-DC interpretation such as cervicitis, severe atrophic changes or difficult-to-diagnose abnormalities. Results classified as inadequate included those where the visible ectocervix was negative for acetowhite changes, but the provider could not visualize the entire squamocolumnar junction (SCJ).

Women with VIA-DC-positive screens were considered eligible for cryotherapy treatment if the cervical lesion met all of the following criteria outlined in WHO guidelines [32, 35]:

1. Complete visualization of transformation zone (TZ) and acetowhite lesion (AWL) margins
2. AWL occupies <75% of the TZ and can be covered by the cryoprobe
3. No suspicion of cancer
4. No evidence of reproductive tract infection or pregnancy

With patient consent, cryotherapy eligible women were offered immediate treatment. Women who did not receive same day cryotherapy were given appointments to follow up for treatment. Clinicians had the option to use free text to record an explanation for lack of same day treatment. Women with cryotherapy ineligible AWL were referred to CBCHS hospitals for treatment with loop electrosurgical excision procedure (LEEP) if they met all of the following criteria:

1. AWL extends into canal or covers >75% of TZ or cannot be covered by cryoprobe
2. No suspicion of cancer
3. No evidence of reproductive tract infection or pregnancy

Biopsies were taken in cases of uncertainty or for lesions that were suspicious for ICC. Biopsy and LEEP specimens underwent histopathologic assessment by pathologists at Yaoundé Gyneco-Obstetric and Pediatric Hospital or Buea Regional Hospital. The histology results were reported according to the Richart CIN staging system [36]. Nurses communicated histopathologic results to patients and helped to arrange appropriate follow-up treatment.

Cervical cancer screening was offered to women aged ≥25 years and to women less than age 25, if they were HIV positive. Pregnant women were excluded.

The CBCHS WHP has minimal external funding and no governmental funding. Thus low cost fees, stratified based on the financial status of the community being served, were charged for screening and treatment in order to sustain the program. Often, women who could not afford to pay were screened and treated at no charge or asked to pay later. The WHP offered additional services including clinical breast exam, family planning and treatment of reproductive tract infection.

### Setting

Screening was conducted at seven WHP sites in hospitals or health centers located in North West, Littoral, Central and South West regions of Cameroon, encompassing urban (Mboppi Baptist Hospital, Etoug-Ebe Baptist Hospital Yaounde, Nkwen Baptist Health Center), semi-urban (Baptist Hospital Mutengene, Banso Baptist Hospital, Kumba Baptist Health Center), and rural referral (Mbingo Baptist Hospital) areas. The catchment area was further broadened through short-term outreach screening clinics.

### Data Collection

Nurses or peer educators collected patient data using a structured, paper-based, clinical record. All patient demographic, social, and medical history, including HIV-status, was self-reported. For biopsy and LEEP specimens, a pathology request form was filled at the time of collection and then the Richart CIN histopathologic diagnosis was recorded by a pathologist at the time of reading [36]. All paper records were sent to the CBCHS Regional Training Center in Mutengene for entry into a centralized electronic database. Clinical data from March 2007 through December 2014 were securely transferred to UMMS Department of Quantitative Health Sciences for analysis. Data on inadequate results was not available from 2007 through 2008, because the WHP enrollment form used during those years did not contain an option to report inadequate results. Data from March 2007 through December 2013 did not include detailed information regarding eligibility for same day treatment. In January 2014, the clinical record forms were updated to capture this information. We performed a secondary analysis of data from January 2014 to December 2014 in order to report same day cryotherapy rates. Histopathology data were only available for women screened from 2007 through 2013 and were not linked to VIA-DC screening data due to inconsistencies in recording of unique patient identifiers. Instances of duplicate patient identifiers were removed from the dataset.

### Statistical Analysis

We obtained summary measures (median, proportions, inter-quartile range) for all characteristics for the total sample as well as by VIA-DC status (positive, negative). Kappa statistic demonstrated that there was a strong agreement between VIA-DC and VILI-DC (*Κ* = 0.927, *p* ≤ 0.001); thus only VIA-DC outcomes were used for analysis. In order to identify factors associated with VIA-DC-positive versus VIA-DC-negative screen, we obtained adjusted odds ratios (AOR) and 95% confidence intervals (CI) from a multivariable logistic regression model with an exchangeable correlation structure to account for the clustering of patients within sites. The covariates included self-reported HIV status (positive, negative, unknown), age (18–29, 30–39, 40–49, 50+), years of education (<8, 8+), marital status (married, single, widowed/separated/divorced), occupation (housewife, formal sector, informal sector, other), screening location (urban, semi-urban, rural referral, mobile clinics), gravidity (0, 1–4, 5+), age at sexual debut (<16, 16–20, 21+) and number of lifetime sexual partners (<2, 2–4, 5+). Two-sided *p<*0.05 was considered to be significant for all tests. Due to low sample size, only descriptive statistics regarding histologic grade of the tissue specimens are presented. ICC prevalence was not calculated for women of unknown age because histopathology data and VIA-DC screening data were not linked due to inconsistency in recording of unique patient identifiers. Cases missing age/date of birth were not consistent between the two datasets. Statistical analysis was conducted using SPSS Version 22.0.

## Results

### Prevalence of VIA-DC-positive screen

**Fig 1:**
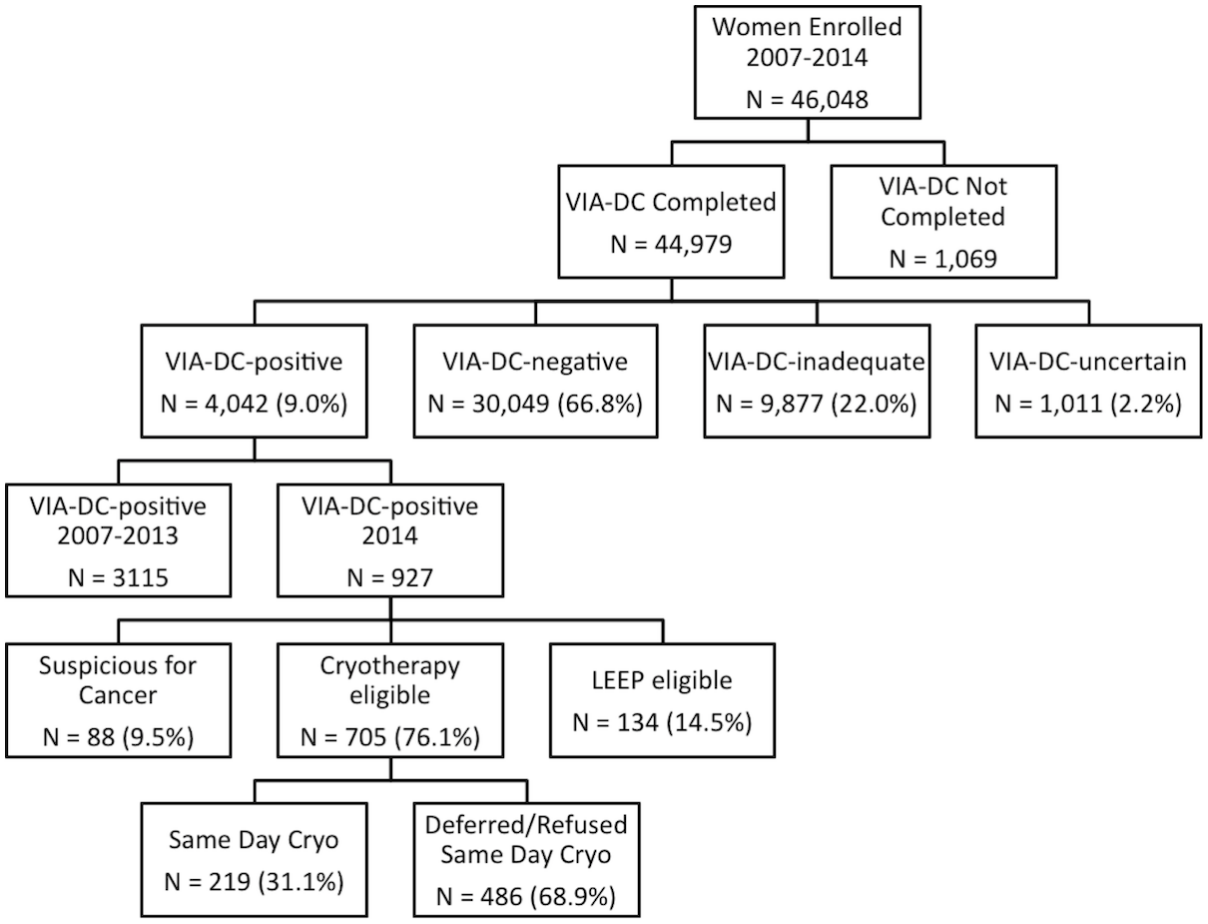
VIA-DC Screening and Treatment Data Flow Chart.

From March 2007 through December 2014 a total of 46,048 women visited the WHP clinics for health services, and 97.7% (n = 44,979) underwent VIA-DC screening (Fig 1). Demographic data is presented in Table 1. The overall VIA-DC-positive rate was 9.0%. The majority of women who underwent cervical cancer screening were VIA-DC-negative (66.8%). A substantial minority (22.0%) screened VIA-DC-inadequate due to incomplete visualization of the transformation zone; annual rates varied from 38.1% to 13.1%. The rate of VIA-DC-inadequate results also varied by age, with women over age 50 having the highest rates of inadequate screens (48.8%) (Table 2). An additional 2.2% of women screened VIA-DC-uncertain due to concurrent reproductive tract infections, severe atrophic changes or difficult-to-diagnose abnormalities that confounded VIA-DC interpretation.

**Table 1:** Characteristics of Women Enrolled in CBCHS WHP Cervical Cancer Screening Program from 2007 to 2014.

Characteristic	Data from 2007–2014
Age, median (IQR)	38 (30–47)
Education (years of school completed), n (%)	
<8	23,546 (55.4)
8+	18,974 (44.6)
Marital Status, n (%)	
Married	29,698 (66.4)
Single (never married)	7,073 (15.8)
Widowed/Separated/Divorced	7,942 (17.8)
Occupation ^a^, n (%)	
Housewife	5,098 (11.8)
Formal Sector	6,031 (13.9)
Informal sector	27,528 (63.5)
Other	4,704 (10.8)
Screening Location, n (%)	
Urban	10,567 (23.6)
Semi-Urban	10,470 (23.4)
Rural Referral	3,932 (8.8)
Mobile Clinics	19,831 (44.3)
Gravidity, n (%)	
0	3,262 (7.4)
1–4	19,141 (43.2)
5+	21,922 (49.5)
Age at Sexual Debut, n (%)	
<16	7,621 (19.0)
16–20	27,425 (68.4)
21+	5,066 (12.6)
Number of Sexual Partners (lifetime), n (%)	
<2	12,445 (30.5)
2–4	19,707 (48.3)
5+	8,623 (21.1)
VIA-DC, n (%)	
VIA-DC-negative	30,049 (66.8)
VIA-DC-positive	4,042 (9.0)
VIA-DC-uncertain	1,011 (2.2)
VIA-DC-inadequate	9,877 (22.0)
Self reported HIV Status, n (%)	
HIV-negative	29,475 (65.5)
HIV-positive	4,933 (11.0)
HIV-unknown	10,571 (23.5)

^a^ Formal occupations: teacher, healthcare worker, secretary; Informal occupations: farmer, trader, seamstress, domestic worker, hair dresser; Other occupations: sex worker, laborer, security officer, military, retired, etc.

**Table 2:** Age-specific VIA-DC Results.

Age Category, n (%)	VIA-DC-negative	VIA-DC-positive	VIA-DC-Inadequate	VIA-DC-Uncertain	Total
18–29	8,138 (77.3)	1,090 (10.4)	1,111 (10.6)	184 (1.7)	10,523
30–39	10,225 (75.1)	1,434 (10.5)	1,669 (12.3)	292 (2.1)	13,620
40–49	7,181 (67.6)	913 (8.6)	2,295 (21.6)	240 (2.3)	10,629
50+	3,987 (42.7)	537 (5.7)	4,561 (48.8)	253 (2.7)	9,338
Unknown	518 (59.6)	68 (7.8)	241 (27.7)	42 (4.8)	869
Total	30,049 (66.8)	4,042 (9.0)	9,877 (22.0)	1,011 (2.2)	44,979

### Predictors of VIA-DC-positive screen

HIV-positive women were 1.36 (95%CI: 1.22–1.53) times more likely than HIV-negative women to screen VIA-DC-positive (Table 3). Women with 8 or more years of education were 10% less likely to screen positive (OR: 0.90, 95% CI: 0.82–0.98) than those who were less educated. Higher gravidity and risk factors related to high-risk sexual behavior, including younger age at sexual debut and increased number of lifetime sexual partners, were independently associated with VIA-DC-positive screen after adjusting for other covariates. Age, marital status and occupation were not associated with VIA-DC-positive screen.

**Table 3:** Adjusted Odds Ratios and 95% Confidence Intervals for VIA-DC-positive Screen.

Variables	VIA-DC-negative	VIA-DC-positive	AOR^a^ [95% CI]	p-value
HIV Status, n (%)				≤.001*
HIV-Negative	21,039 (70.0)	2,526 (62.5)	1	
HIV-Positive	3,209 (10.7)	682 (16.9)	1.36 [1.22–1.53]	
HIV-Status Unknown	5,801 (19.3)	834 (20.6)	1.18 [1.07–1.30]	
Age, n (%)				.216
≤29	8,138 (27.6)	1,090 (27.4)	1	
30–39	10,225 (34.6)	1,434 (36.1)	0.99 [0.89–1.09]	
40–49	7,181 (24.3)	913 (23.0)	0.91 [0.81–1.02]	
50+	3,987 (13.5)	537 (13.5)	0.89 [0.76–1.03]	
Education, n (%)				.020*
<8	14,874 (52.0)	2,080 (54.5)	1	
8+	13,739 (48.0)	1,740 (45.5)	0.90 [0.82–0.98]	
Marital Status, n (%)				.294
Married	20,768 (69.5)	2,615 (65.1)	1	
Single (never married)	5,032 (16.8)	721 (17.9)	1.07 [0.95–1.19]	
Widowed/Separated/Divorced	4,069 (13.6)	682 (17.0)	1.08 [0.96–1.20]	
Occupation, n (%)				.758
Housewife	3,569 (12.3)	450 (11.7)	1	
Formal	4,307 (14.8)	511 (13.3)	1.00 [0.86–1.17]	
Informal	17,715 (61.0)	2,436 (63.6)	1.04 [0.93–1.18]	
Other	3,453 (11.9)	433 (11.3)	1.07 [0.91–1.26]	
Age at Sexual Debut, n (%)				.003*
<16	4,904 (18.4)	746 (21.2)	1	
16–20	18,398 (69.1)	2,407 (68.4)	0.91 [0.83–0.99]	
21+	3,337 (12.5)	365 (10.4)	0.78 [0.68–0.90]	
Number of Sexual Partners (lifetime), n (%)				≤.001*
<2	7,808 (28.9)	848 (23.8)	1	
2–4	13,457 (49.9)	1,800 (50.4)	1.15 [1.04–1.26]	
5+	5,720 (21.2)	921 (25.8)	1.33 [1.19–1.49]	
Gravidity, n (%)				.002*
0	1,797 (7.7)	151 (3.9)	1	
1–4	11,156 (47.9)	2,397 (62.3)	1.32 [1.21–1.56]	
5+	10,333 (44.4)	1,299 (33.8)	1.23 [1.03–1.48]	

^a^Obtained from multivariable logistic regression model that included all variables in the table and adjusted for the clustering of patients within sites.

* Two-sided *p<*0.05 was considered to be significant.

### Treatment uptake

Women who screened VIA-DC-positive were offered same day cryotherapy, biopsy or referral for LEEP depending upon the characteristics and severity of dysplasia of the observed lesion. Data from 2007 through 2013 did not include detailed information regarding eligibility for same day treatment, rates of uptake, or reasons for attrition. In 2014, the data collection form was updated to document eligibility for cryotherapy based on the previously described WHO guidelines. In 2014 alone, 12,191 women underwent VIA-DC screening, and 927 (7.6%) screened VIA-DC-positive (Fig 1). Of these women, 705 (76.0%) were eligible for same day cryotherapy, 219 (31.1%) underwent same day treatment, and 486 who did not receive same day treatment were asked to return for follow up (Fig 1). Of the 486 women who did not undergo same day treatment, 54 records (11.1%) included notation regarding reason for delay in treatment. Of those records, 14.8% (n = 8) cited equipment failure (empty carbon dioxide tank, cryotherapy probe out for repair), 25.9% (n = 14) cited desire to discuss treatment with husband/family, 24.1% (n = 13) cited referral for LEEP due to co-morbid genital condyloma, other complicating conditions or patient preference to wait until treatment of another condition was resolved, 33.3% (n = 18) cited deferment of cryotherapy until review of cervicographs by a supervisor, and 1.9% (n = 1) cited inability to pay for procedure. Women who screened VIA-DC-positive, but required LEEP (n = 134) or underwent biopsy due to suspicion for cancer (n = 88) were not eligible for same day treatment. The proportion of women who returned for treatment as counseled is unknown.

### Prevalence of invasive cervical cancer

From March 2007 through December 2013, 32,788 women were screened with VIA-DC and 764 specimens were collected from women with VIA-DC-positive and -uncertain screens. Among biopsy and LEEP specimens 14 (1.8%) were negative, 246 (32.2%) were CIN1, 177 (23.2%) were CIN2, 126 (16.5%) were CIN3/carcinoma-in-situ and 201 (26.3%) were ICC (Table 4). Assuming that VIA-DC screening captured all ICC cases among women who visited the WHP for screening from 2007 through 2013, the prevalence was 613 per 100,000 women screened. The majority of ICC cases were in women greater than 50 years and the prevalence of ICC among women screened also increased with age to a high of 1,717 per 100,000 in the over 50 age group (Table 4).

**Table 4:** Frequency and Age-specific Rates^a^ of Histopathologic Results from Biopsy and LEEP specimens.

Age Group	Total VIA-DC Screened 2007–13	Total Specimens	Negative, n (%)	CIN1, n (%)	CIN2, n (%)	CIN3, n (%)	ICC, n (%)	ICC Prevalence
18–29	7,822	122	1 (0.80)	69 (56.6)	34 (27.9)	12 (9.8)	6 (4.9)	77
30–39	10,098	221	3 (1.4)	74 (33.5)	79 (35.7)	47 (21.3)	18 (8.1)	178
40–49	7,635	153	3 (2.0)	53 (34.6)	37 (24.2)	22 (14.4)	38 (24.8)	498
50+	6,464	182	3 (1.6)	27 (14.8)	14 (7.7)	27 (14.8)	111 (61.0)	1,717
Unknown	769	86	4 (4.7)	23 (26.7)	13 (15.1)	18 (20.9)	28 (32.6)	-
Total	32,788	764	14 (1.8)	246 (32.2)	177 (23.2)	126 (16.5)	201 (26.3)	613

^a^Rates per 100,000 women screened

## Discussion

The CBCHS WHP operates the largest cervical cancer screening program in Cameroon, a country with the highest HIV prevalence in Western and Central Africa and where ICC is a major contributor to cancer mortality [2, 6]. From 2007 through 2014, 44,979 women, nearly all (97.7%) of those attending WHP clinics, were assessed through a fee-for-service visual inspection screening program. Using this data we i) determined prevalence (9.0%) and predictors of VIA-DC-positive screen (HIV-positivity, young age at sexual debut, higher number of lifetime sexual partners, low education status, higher gravidity), ii) documented rates of same day cryotherapy treatment in 2014 (31.1%), and iii) described the cohort ICC prevalence (613 per 100,000 women screened). Given the large number of women screened and the eight-year data collection period, this analysis provides new and robust insights into the magnitude of cervical pre-cancer and cancer in Cameroon.

### Prevalence of VIA-DC-positive screen

The prevalence of VIA-DC-positive screen from 2007 through 2014 was 9.0%. These findings are lower than or similar to two previous studies conducted in Cameroon. Specifically, one study performed from 2001 to 2002 screened 4,813 women, comparing VIA and cytology as screening methods for CIN/ICC, and reported a VIA-positive rate of 21.7%, higher than our finding [28]. A possible explanation for inconsistency in rates between the studies is difference in cohort risk for VIA-positive screen. The aforementioned study lacked risk factor characterization of the sample, for example they did not report HIV-status or lifetime number of sexual partners. If their sample was at increased risk for VIA-positive screen we would expect a higher VIA-positive rate. Another small screening program conducted from 2010 through 2012 evaluated the effectiveness of HPV testing on self-obtained specimens followed by sequential VIA testing and reported a VIA-positive rate of 12.9%, similar to our finding [27]. HIV status was not reported; however, other VIA-positive risk factors were comparable between the studies. Though lower than some previous studies, a rate of 9% VIA-DC-positive screen in a cohort of 44,979 women, stable over a period of eight years, suggests a significant burden of potential cervical cancer cases and highlights the need for expansion of cervical cancer screening and prevention throughout the 10 regions of Cameroon.

The rate of VIA-DC-uncertain screens due to suspected cervicitis, severe atrophic changes or difficult-to-diagnose abnormalities that confounded evaluation of the ectocervix was 2.2% and is overall consistent with other reports in African countries, including Botswana [15], Kenya [13], Côte d'Ivoire [12] and Zambia [37]. Other SSA studies did not report rates of VIA-confounded results [19, 23].

The rate of VIA-DC-inadequate screens was high (22.0%). Although in these cases the ectocervix was negative, the clinician was unable to visualize the entire SCJ. Most screening programs do not include a VIA-DC-inadequate category. For example the CCPPZ classification includes only negative, positive, suspicious for cancer and VIA-uncertain/indeterminate which is designated for those with non-neoplastic cervical abnormalities confounding VIA-DC interpretation such as cervicitis or severe atrophic changes. Thus the CCPPZ classifies a negative ectocervix with a non-visible SCJ as negative [37] whereas the WHP classifies it as VIA-DC-inadequate.

The only study that included a category comparable to VIA-DC-inadequate was conducted in Côte d'Ivoire and documented a rate of 2.3% [12]. The lower rate of inadequate screens in the Côte d'Ivoire study is likely due to two factors. First, the women in the Côte d'Ivoire study were younger (Median age = 36, IQR: 31–42 for Côte d'Ivoire vs Median age = 38, IQR: 30–47 for CBCHS) and thus less affected by the normal cervical aging process which includes squamous epithelialization of the cervix that migrates the SCJ up into the endocervical canal [34]. Second, the Côte d'Ivoire study used VIA, not VIA-DC. VIA-DC incorporates a camera into the screening process that, in some cases, complicates or precludes manipulation of the cervix with a cotton tipped applicator to view the entire SCJ. Internal evaluation of the WHP screening process revealed that some portion of VIA-DC-inadequate results was due to lack of technical expertise among trained nurses at manipulating the cervix. For example, the annual VIA-DC-inadequate rate peaked at 38.1% when senior staff were pulled away from screening clinics to work on CBCHS WHP’s successful Gardasil HPV vaccination campaign [38]. Following staff training in techniques to expose the SCJ the VIA-DC-inadequate rate dropped to 13.1%, a rate similar to inadequate results documented in colposcopy, a technique that has similar limitations [39].

In part due to the complications mentioned above, the WHO does not prioritize VIA screening for women over age 50 [32]. In our program, however, a substantial number of women over age 50 screened VIA-DC-positive (5.7%), and they accounted for 152 (30.2%) of all 504 histopathologically confirmed CIN2+ cases. While the rates of VIA-DC positivity were lower for the over 50 age group than any other age group, the prevalence of ICC was higher than any other age group (1,717 per 100,000 women screened). Thus, screening in this age group may provide meaningful clinical benefit, especially if cancer treatment is available. Training clinicians on techniques to better visualize the SCJ in older patients and utilizing an additional screening method such as HPV co-testing, when VIA-DC-inadequate results are found are possible ways to decrease the number of VIA-DC-inadequate screens and better inform treatment decisions.

### Predictors of VIA-positive screen

Previous studies have shown that risk factors for VIA-positive screen in developing countries include HIV-positive status [12, 13], low level of education, high number of lifetime sexual partners, and younger age at sexual debut [37]. Our findings echo these results and indicate that targeting screening toward women in Cameroon with these high risk characteristics may prove particularly beneficial. The association of age with VIA-positive screen varies; some studies have reported lower rates of VIA-positivity with increasing age [13, 37], others have reported the opposite [17]. In our study, there was a non-significant trend towards lower rates of VIA-positivity with increasing age. A large proportion (48.8%) of women aged over 50 were classified as VIA-DC-inadequate rather than VIA-DC-negative due to incomplete visualization of the SCJ and this may confound the association with age.

### Treatment uptake

The WHO screen-and-treat approach is recommended for cervical cancer prevention in developing countries due to its efficacy in decreasing cervical cancer related morbidity and mortality [32, 40]. In addition to low cost, distinct advantages of this method include decreasing the number of patients who are lost to follow up and minimizing cost incurred as a result of needing to recall patients to the clinic. Despite offering same day treatment at all sites, the rate of same day cryotherapy among women who screened VIA-DC-positive at the WHP was only 31.1% in 2014. Rates of same day cryotherapy have been reported to be higher in Zambia (65%) [41], Ghana (70.2%) [11], and India (74.8%) [16]. These studies have reported barriers to uptake of same day treatment including need to seek permission from spouse and equipment failure or unavailability [11, 16]. Although we did not quantitatively measure explanations for low utilization of same day treatment, retrospective review of staff notations in the medical records revealed similar reasons. An additional barrier to same day treatment in the WHP was cost. Although only a small percentage of staff notations reported cost as a barrier to same day cryotherapy, nurses were not required to record a reason for non-treatment and consequently this barrier is likely underreported. Informal discussion with WHP nurses regarding reasons for same day cryotherapy refusal included cost as a major factor. Our evaluation highlights that a VIA-DC-positive screen does not result in reflexive uptake of same day cryotherapy, especially in the face of health system challenges (equipment failure was reported by 14.8% while 33.3% reported need for review of cervicogragraphs by a supervisor who may not have been immediately available), cultural demands (25.9% reported need to discuss treatment with family members) and separate payment for the procedure. These factors undermine the WHO-endorsed screen-and-treat approach in this setting and intimate that strengthening the health system and restructuring the payment scheme might improve same day treatment of VIA-DC-positive lesions.

### Prevalence of invasive cervical cancer

The 5-year age-standardized prevalence of ICC in Cameroon, as estimated by GLOBOCAN 2012 analysis from the IARC, is 80.7 per 100,000 [2]. Due to the absence of a national cancer registry and low quality of source data, current GLOBOCAN estimates may not accurately reflect the ICC burden in Cameroon [29, 30]. Despite its limitations discussed below, in the absence of a robust and rigorous dataset from which population-based ICC prevalence is calculated, data from a large-scale screening program such as the CBCHS WHP may provide additional insight into the burden of ICC in Cameroon.

The prevalence of ICC in women screened by the CBCHS WHP from 2007 through 2013 was 613 per 100,000. This result is not age-standardized and therefore not comparable with population level data from GLOBOCAN. Nevertheless, the cohort specific ICC prevalence is high. This is likely due in part to selection bias; women who self-presented or who were referred to cervical cancer screening were more likely to have gynecologic symptoms or risk factors for cervical cancer and thus do not represent the general population. In contrast, some women who presented for VIA-DC screening and had lesions eligible for LEEP or biopsy did not receive the recommended procedure and therefore we do not know their true cervical cancer status. Our analysis assumes that these women are cancer-free, but some percentage of these women may have had ICC. In this respect, the cohort prevalence of ICC reported in this manuscript may underestimate the true prevalence of ICC in Cameroon.

Though high, the WHP prevalence of ICC was lower than that reported by the CCPPZ (700 per 100,000). This may be due in part to the difference in self reported HIV prevalence between the two screened groups (11% HIV-positive in Cameroon vs 28% in Zambia) [41], different prevalence of HIV/AIDS nationally (4.5% vs. 12.7% in 2012) [6] and different rates of male circumcision (94% vs. 21.6%) [42, 43] in Cameroon vs. Zambia as it has been shown that HIV-HPV co-infection may play a synergistic role in development of cervical cancer [44] and that male circumcision reduces the risk of HPV infection substantially [45, 46].

The prevalence of ICC was associated with age, peaking in women aged over 50. This finding underscores the need for data regarding outcomes of treatment in women diagnosed with ICC in resource poor settings in order to determine whether screening women aged 50 and over is worthwhile.

### Limitations

This analysis was a retrospective evaluation of a large-scale, grassroots, fee-for-service cervical cancer screening program. Although this serves as a strength because it provides striking insight into the realities of implementing a nearly self-sustaining program that did not rely on short-term grant funding or expert oversight from a research team, there are limitations that should be addressed to improve the quality of care. Some VIA-DC result categories changed over time and in some cases were not comparable to categories in other studies. The high rate of VIA-DC-inadequate results was in part dependent upon clinician expertise in VIA-DC technique. The HIV status of our patient population was self-reported. The data available regarding treatment uptake is limited. Outcome of treatment decision (same day cryotherapy versus referral) was systematically documented beginning in 2014, but reasons for treatment choice were not systematically reported, leaving the question of specific barriers to treatment answerable only by anecdotal evidence per nurse clinician notes and informal discussion with WHP staff. Data regarding follow-up treatment of cryotherapy eligible lesions was also not available, precluding calculation of attrition rate among women referred for treatment at a later date.

At the time of data analysis, histopathology data was available only for women screened from 2007 through 2013. We believe this delay is an honest reflection of the logistical difficulty of pathologic diagnosis, dissemination of findings and upkeep of an electronic database in this low-resource setting. Since ICC prevalence was calculated from women who self-presented for screening or were referred by other providers, selection bias may have caused over-estimation of the true prevalence. Additionally, removal of cases due to instances of duplicate patient identifiers and lack of pathology specimens on VIA-DC-positive and -uncertain women who did not consent to biopsy or LEEP and were thus lost to follow-up may have caused under-estimation of the true ICC prevalence.

## Conclusion

The high rate of VIA-DC-positive results in Cameroon suggests a significant impending burden of ICC cases. The rate of VIA-DC-inadequate (normal ectocervix, but portions of the transformation zone were obscured) screens was high, especially in women aged 50 and over. Training clinicians on techniques to better visualize the SCJ and adding an additional screening method to the protocol in cases of VIA-DC-inadequate results are possible ways to confirm whether these cases are truly negative. In comparison to similar screening programs in SSA, we report low utilization of same day cryotherapy treatment. Further studies are required to characterize possible program specific barriers to treatment, for example cultural demands, health system challenges and cost of procedure. The prevalence of ICC among women who presented for screening was high and requires further investigation.
